# Generation of an immortalized mouse embryonic palatal mesenchyme cell line

**DOI:** 10.1371/journal.pone.0179078

**Published:** 2017-06-05

**Authors:** Katherine A. Fantauzzo, Philippe Soriano

**Affiliations:** 1 Department of Cell, Developmental and Regenerative Biology, Icahn School of Medicine at Mount Sinai, New York, New York, United States of America; 2 Department of Craniofacial Biology, School of Dental Medicine, University of Colorado Anschutz Medical Campus, Aurora, Colorado, United States of America; Medical University of South Carolina, UNITED STATES

## Abstract

Palatogenesis is a complex morphogenetic process, disruptions in which result in highly prevalent birth defects in humans. In recent decades, the use of model systems such as genetically-modified mice, mouse palatal organ cultures and primary mouse embryonic palatal mesenchyme (MEPM) cultures has provided significant insight into the molecular and cellular defects underlying cleft palate. However, drawbacks in each of these systems have prevented high-throughput, large-scale studies of palatogenesis *in vitro*. Here, we report the generation of an immortalized MEPM cell line that maintains the morphology, migration ability, transcript expression and responsiveness to exogenous growth factors of primary MEPM cells, with increased proliferative potential over primary cultures. The immortalization method described in this study will facilitate the generation of palatal mesenchyme cells with an unlimited capacity for expansion from a single genetically-modified mouse embryo and enable mechanistic studies of palatogenesis that have not been possible using primary culture.

## Introduction

The complex morphogenetic process of mammalian craniofacial development begins with the formation of five facial prominences, including the frontonasal prominence, a pair of maxillary prominences and a pair of mandibular prominences. Subsequent formation of the nasal pits divides the frontonasal prominence into the medial and lateral nasal processes, which will in turn fuse to give rise to the nostrils. A second fusion event between the medial nasal processes and the maxillary prominences forms the upper lip, at which time the secondary palatal shelves first appear as morphologically distinct outgrowths on the oral side of the maxillary prominences. As the shelves extend from the maxillae, they grow downward such that they are positioned on either side of the tongue. With development of the jaw, the palatal shelves elevate to a horizontal, apposing position above the tongue and grow towards the midline. Upon meeting, the palatal shelves fuse with one another and eventually with two derivatives of the medial nasal processes, the primary palate anteriorly and the nasal septum superiorly, resulting in a continuous palate that separates the oral and nasal cavities. Palatogenesis begins at E11.5 in the mouse and posterior palatal fusion is complete by E16.5, while these same processes occur between 6–12 weeks of gestation in humans. Following fusion, the anterior mesenchyme of the secondary palate undergoes intramembranous ossification to generate the bones of the hard palate, while the posterior secondary palate gives rise to the muscular soft palate [1].

Disruption of any of the sequential events necessary for proper palatogenesis can result in a cleft palate [1]. Defects in craniofacial development, including cleft lip and palate, comprise one of the most prevalent birth defects in humans. The National Center on Birth Defects and Developmental Disabilities recently estimated that each year in the United States alone there are 2,651 live births with cleft palate (1 in 1,574 births) and 4,437 live births with cleft lip with or without a cleft palate (1 in 940 births) [2]. Cleft palate often requires surgical intervention early in life, which can result in long-term complications such as dental caries, maxillary hypoplasia, dental arch deformities, fistulas and velopharyngeal insufficiency [3,4].

In recent decades, the use of model systems has shed considerable light on the molecular and cellular defects underlying cleft palate. Given the remarkable anatomical similarities between secondary palatogenesis in humans and mice, the majority of this data has been obtained from *in vivo* studies of genetically-modified mouse models [1,5]. Additional *in vitro* models such as mouse palatal organ cultures and primary mouse embryonic palatal mesenchyme (MEPM) cell cultures [6–9] have allowed researchers to systematically test the effect(s) of altering the concentrations of exogenous, soluble factors, such as growth factors and chemical inhibitors, on palatal cell activity, gene expression and protein phosphorylation [10–14]. Primary MEPM cells are a particularly appealing model for molecular pathway dissection, as passaging of these cultured cells multiplies the number of mesenchymal cells that can be derived from each palatal shelf pair. Further, unlike treatment of multi-layered palatal organ cultures, primary MEPM cells are cultured as a monolayer, thereby ensuring uniform distribution of exogenous factors across the cells. However, slight variations in embryo age and dissection technique between primary MEPM derivations likely result in subtle changes in culture characteristics. Furthermore, primary MEPM cells are split at the relatively low ratio of 1:3 through 2–3 passages, at which point their proliferation rate dramatically decreases, hence limiting the expansion that would be required for large-scale studies. These drawbacks thus necessitate the development of a homogeneous, well-defined MEPM cell line with increased proliferative potential.

Over twenty years ago, *Cdkn2a*-null mice were generated [15], which lack the products of both alternative splice transcripts from the *Cdkn2a* locus, cyclin-dependent kinase inhibitor 2A (Cdn2a; also known as p16-INK4a) and tumor suppressor ARF (Arf; also known as p19-ARF) [16]. Primary mouse embryonic fibroblasts (MEFs) derived from *Cdkn2a*^-/-^ embryos, while morphologically indistinguishable from their wild-type counterparts, exhibited accelerated growth, higher cellular densities and increased colony forming efficiency at low passages. Moreover, serial passaging of these *Cdkn2a*-null MEFs produced sustained growth through 25 passages with no apparent senescent phase, unlike wild-type MEFs [15]. In addition to fibroblasts, a wide range of immortalized cell types have been generated from *Cdkn2a*^*-/-*^ embryos, such as keratinocytes, melanocytes, glia, lymphocytes and macrophages [17–20], indicating that this method of immortalization is applicable to a diverse array of model systems.

Here, we detail the generation of an immortalized MEPM cell line with similar morphology, migration ability, marker expression and biochemical properties to primary MEPM cells. Given the reduced variability of these cells compared to their primary counterparts and their unlimited capacity for expansion, use of this cell line has the potential to accelerate large-scale modeling of palatogenesis *in vitro*. Further, introduction of the *Cdkn2a*^*-*^ allele into genetically-defined mouse models with established craniofacial defects should facilitate the modeling of mechanisms underlying the etiology of cleft palate.

## Materials and methods

### Mouse strains

All animal experimentation was approved by the Institutional Animal Care and Use Committee of Icahn School of Medicine at Mount Sinai. Wild-type and *Cdkn2a*^*+/tm1Rdp*^ mice [15], referred to in the text as *Cdkn2a*^*+/-*^, were maintained on a 129S4 coisogenic genetic background. Mice were euthanized by inhalation of carbon dioxide from compressed gas. Cervical dislocation was used as a secondary verification of death.

### Cell culture

Primary MEPM cells were isolated from the palatal shelves of wild-type embryos dissected at E13.5 (day of plug considered 0.5 days) in 1x phosphate buffered saline (PBS) and cultured on plastic dishes in medium (Dulbecco’s modified Eagle’s medium (GIBCO, Invitrogen, Carlsbad, CA, USA) supplemented with 50 U/mL penicillin (GIBCO), 50 μg/mL streptomycin (GIBCO) and 2 mM L-glutamine (GIBCO)) containing 10% fetal bovine serum (FBS; HyClone Laboratories, Inc., Logan, UT, USA) as previously described [13]. Primary MEPM cells were split at a ratio of 1:3 through at most three passages. Immortalized MEPM cells were isolated from *Cdkn2a*^*-/-*^ E13.5 embryo palatal shelves as described above and cultured on plastic dishes in medium containing 10% FBS. Immortalized MEPM cells have been split at a ratio of 1:5 through at least 22 passages. Cultured cells were photographed using a Nikon DS-Fi1 color camera (Nikon Instruments Inc., Melville, NY, USA) fitted onto a Nikon Eclipse TS100 inverted microscope (Nikon Instruments Inc.).

### Ki67 immunofluorescence analysis

Cells were seeded onto uncoated glass coverslips. The following day, subconfluent cells were fixed in 4% paraformaldehyde (PFA) in PBS with 0.1% Triton X-100 for 10 min and washed in PBS with 0.1% Triton X-100. Cells were blocked for 1 h in 5% normal donkey serum in PBS and incubated overnight at 4°C in anti-Ki67 primary antibody (1:300; Invitrogen) diluted in 1% normal donkey serum in PBS. After washing in PBS, cells were incubated in Alexa Fluor 488-conjugated donkey anti-rabbit secondary antibody (1:1,000; Invitrogen) diluted in 1% normal donkey serum in PBS with 2 μg/mL 4’,6-diamidino-2-phneylindole (DAPI; Sigma-Aldrich Corp., St. Louis, MO, USA) for 1 hr. Cells were mounted in Aqua Poly/Mount mounting medium (Polysciences, Inc., Warrington, PA, USA) and photographed using an ORCA-Flash4.0 LT digital camera fitted onto an Axio Imager.M2 fluorescence microscope (Carl Zeiss Microscopy, LLC, Thornwood, NY, USA).

### Alkaline phosphatase staining

Confluent cells were fixed in 4% PFA in PBS for 2 min, washed in PBS and stained in buffer containing 100 mM NaCl, 100 mM Tris HCl pH 9.5, 50 mM MgCl_2_, 0.1% Tween 20, 250 μg/mL 4-Nitro blue tetrazolium (NBT) and 125 μg/mL 5-bromo-4-chloro-3-indolyl phosphate, 4-toluidine salt (BCIP) for 30 min. Cells were photographed in PBS using an Axiocam 105 color camera fitted onto a Stemi 508 stereo microscope (Carl Zeiss Microscopy, LLC).

### Scratch assays

Cells were seeded onto glass coverslips coated with 5 μg/mL human plasma fibronectin purified protein (EMD Millipore Corporation, Billerica, MA, USA). At ~90–100% confluence, cells were scratched with a P1000 pipet tip, washed with PBS and incubated in fresh medium containing 10% FBS for 6 hr. Cells were subsequently processed for immunofluorescence analysis as detailed above using anti-paxillin primary antibody (1:250; Y113; Abcam Plc, Cambridge, MA, USA) with rhodamine-conjugated phalloidin (1:600; Biotium, Inc., Fremont, CA, USA). Cells were photographed using an Olympus DP71 digital camera (Olympus America Inc., Waltham, MA, USA) fitted onto an Olympus BX51 fluorescence microscope (Olympus America Inc.).

### Transwell assays

Cells were serum-starved for 24 hr in medium containing 0.1% FBS. Cell culture inserts for 24-well plates containing polyethylene terephthalate membranes with 8 μm pores (Corning Inc., Corning, NY, USA) were coated with 5 μg/mL human plasma fibronectin purified protein (EMD Millipore Corporation). Cells were seeded at a density of 315,000 cells per insert in 250 μL medium containing 0.1% FBS and inserts were immersed in 500 μL medium containing 10% FBS supplemented with 10 ng/mL PDGF-AA for 4 hr. Migrated cells were subsequently fixed in 4% PFA in PBS for 10 min and stained in 0.1% crystal violet in 10% ethanol for 10 min. Dried inserts were photographed using an Axiocam 105 color camera fitted onto a Stemi 508 stereo microscope (Carl Zeiss Microscopy, LLC). Five fields of cells from each of three independent trials were photographed and quantified by measuring integrated density with ImageJ software (version 1.50i; National Institutes of Health, Bethesda, MA, USA). Statistical analyses were performed with Prism 6 (GraphPad Software, Inc., La Jolla, CA, USA) using a two-tailed unpaired *t*-test.

### Quantitative RT-PCR

Total RNA was isolated from cultured cells using the RNeasy Mini Kit (Qiagen Inc., Valencia, CA, USA) according to the manufacturer’s instructions. First-strand cDNA was synthesized using a ratio of 2:1 random primers: oligo (dT) primer and SuperScript II RT (Invitrogen) according to the manufacturer’s instructions. qRT-PCR was performed on a Bio-Rad iQ5 Multicolor Real-Time PCR Detection System and analyzed with iQ5 Optical System Software (version 2.0; Bio-Rad Laboratories, Inc., Hercules, CA, USA). All reactions were performed with PerfeCTa SYBR Green FastMix for iQ (Quanta Biosciences, Inc., Gaithersburg, MD, USA), 300 nM primers (Integrated DNA Technologies, Inc., Coralville, IA, USA) and 100 ng cDNA in a 20 μL reaction volume. The following PCR protocol was used: step 1: 95°C for 3 min; step 2: 95°C for 10 s; step 3: 60°C for 30 s; repeat steps 2 and 3 for 40 cycles; step 4 (melting curve): 55°C for 30 s; repeat step 4 for 81 cycles. All samples were run in triplicate for three independent runs and normalized against an endogenous internal control, *B2m*, using the 2^-ΔΔC^_T_ method [21]. Statistical analyses were performed with Prism 6 (GraphPad Software, Inc.) using a two-tailed unpaired *t*-test. The qRT-PCR primers used can be found in Table 1.

**Table 1:** Primers used in qRT-PCR analysis.

Transcript	Forward primer (5’ to 3’)	Reverse primer (5’ to 3’)
*mB2m*	ACTGACCGGCCTGTATGCTA	TGAAGGACATATCTGACATCTCTA
*mOsr2*	CATTTCGGAGGCAAGATCAC	GTTTCGCCTGAACACTTTGC
*mPdgfra*	GATAGTGGAGAACCTGTTGC	TCAGTCTCTGTTCGTCCAGG
*mMsx1*	GAAAGCCCCGAGAAACTAGA	TAGACAGGTACTGCTTCTGG
*mShox2*	CTATCCAGACGCTTTCATGC	CAAGCTTCAAACTGGCTAGC
*mEfnb1*	TGTCCTGGAGCTCTCTTAAC	GATCAAGCACAGTGCTGCAA
*mMn1*	CATGAGTACTATCGACCTGG	TTGTTGGTGGGGTGGTCATG
*mTbx22*	CTTCTAGGGACAGTCAACAG	AGTGCCAATGTCGTGGAATC
*mMeox2*	GCAGACGTAGAGAAGAGAAG	TATCTTCTCAGTCTGGTCAG
*mAlx3*	CCTATGACATCTCCGTACTG	CATGAAGCCAGCCACATTTC
*mShh*	CCACTGTTCTGTGAAAGCAG	AAGGTGAGGAAGTCGCTGTA

### Immunoprecipitations and Western blotting

To induce PDGF receptor signaling, cells at ~70% confluence were serum-starved for 24 hr in medium containing 0.1% FBS and stimulated with 10 ng/mL PDGF-AA, PDGF-BB or PDGF-DD ligand (R&D Systems, Inc., Minneapolis, MN, USA) for 15 min at 37°C. Cells were harvested in ice-cold lysis buffer (20 mM Tris pH 8, 150 mM NaCl, 10% glycerol, 1% Nonidet P-40, 2 mM EDTA, 1x complete mini protease inhibitor cocktail [Roche Diagnostics, Indianapolis, IN, USA], 1 mM PMSF, 10 mM NaF, 1 mM Na_3_VO_4_, 25 mM β-glycerophosphate) and lysates were collected by centrifugation at 12,000 rpm for 20 min at 4°C. For immunoprecipitations, cell lysates were incubated with PDGFRα (1:500; C-20; Santa Cruz Biotechnology, Inc., Dallas, TX, USA), PDGFRβ (1:500; 958; Santa Cruz Biotechnology, Inc.) or phospho-Ser/Thr Akt substrate (1:175; Cell Signaling Technology, Inc., Danvers, MA, USA) primary antibody overnight at 4°C followed by incubation with 20 μL of protein A-agarose (Santa Cruz Biotechnology, Inc.) for 2 hr at 4°C the following day. Beads were washed with lysis buffer five times and the precipitated proteins were eluted with Laemmli buffer containing 10% β-mercaptoethanol, heated for 5 min at 95°C and separated by SDS-PAGE. Western blot analysis was performed according to standard protocols using horseradish peroxidase-conjugated secondary antibodies (1:10,000; Jackson Immunoresearch Laboratories, Inc., West Grove, PA, USA). The following antibodies were used for Western blotting: PDGFRβ (1:200; 958; Santa Cruz Biotechnology, Inc.), phosphotyrosine (1:1,000; 4G10; EMD Millipore Corporation), PDGFRα (1:200; C-20; Santa Cruz Biotechnology, Inc.), phospho-Akt (1:1,000; Ser473; Cell Signaling Technology, Inc.), Akt (1:1,000; Cell Signaling Technology, Inc.), phospho-p44/42 MAPK (1:1,000; Erk1/2; Thr202/Tyr204; Cell Signaling Technology, Inc.), p44/42 MAPK (1:1,000; Erk1/2; Cell Signaling Technology, Inc.), phospho-(Ser/Thr) Akt Substrate (1:1,000; Cell Signaling Technology, Inc.) and β-tubulin (1:1,000; E7; Developmental Studies Hybridoma Bank, Iowa City, IA, USA).

## Results and discussion

### Immortalized MEPM cells are proliferative, undifferentiated mesenchymal cells

**Fig 1:**
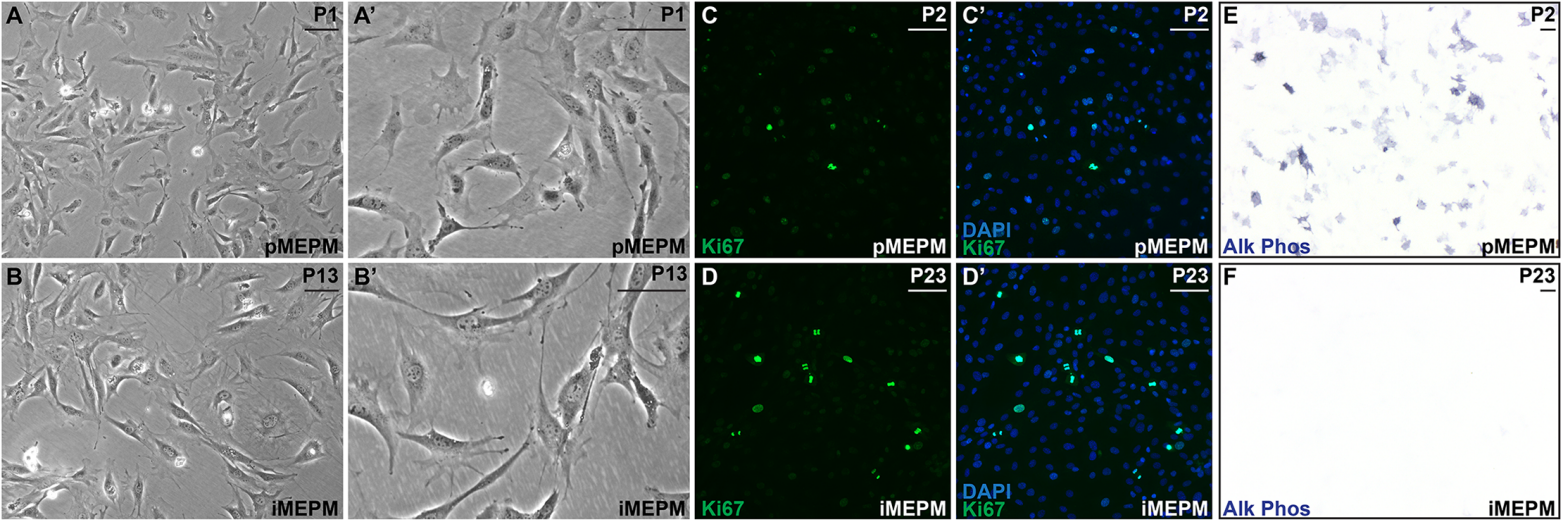
Immortalized MEPM cells are proliferative, undifferentiated mesenchymal cells. (A-B’) Mesenchymal morphology of passage 1 primary (A,A’) and passage 13 immortalized (B,B’) MEPM cells in culture at low (A,B) and high (A’,B’) magnification. (C-D’) Expression of Ki67 (green) as assessed by immunofluorescence analysis in passage 2 primary (C,C’) and passage 23 immortalized (D,D’) MEPM cells. Both primary and immortalized MEPM cells actively proliferate in subconfluent culture. Nuclei were stained with DAPI (blue; C’,D’). (E,F) Alkaline phosphatase staining of passage 2 primary (E) and passage 23 immortalized (F) MEPM cells. A subset of primary MEPM cells express a marker of osteoblast differentiation while immortalized MEPM cells remain undifferentiated. Scale bars, 100 μm.

To generate an immortalized MEPM cell line, the palatal shelves of E13.5 *Cdkn2a*^*-/-*^ embryos were dissected in 1x PBS, minced with forceps and dissociated in 0.25% trypsin/1 mM EDTA for 10 min at 37°C. Cells were subsequently cultured in DMEM supplemented with 50 U/mL penicillin, 50 μg/mL streptomycin, 2 mM L-glutamine and 10% FBS. Upon culturing, both low-passage primary MEPM (pMEPM) cells (Fig 1A and 1A’) and high-passage immortalized MEPM (iMEPM) cells (Fig 1B and 1B’) exhibited a mesenchymal morphology. These cultures consisted of long, thin cells which were broadly dispersed on the plastic substrate. Individual cells had a relatively small cell body, which contained a large, round nucleus with pronounced nucleoli, and numerous cellular processes (Fig 1A–1B’). Further, Ki67 immunofluorescence analysis revealed that both cell types were actively proliferating in subconfluent culture (Fig 1C–1D’). When grown to confluence, a subset of primary MEPM cells expressed the osteoblast marker alkaline phosphatase [22] (Fig 1E), while immortalized MEPM cells remained undifferentiated (Fig 1F). Collectively, these results demonstrate that immortalized MEPM cells are a stable population of proliferative, undifferentiated mesenchymal cells.

### Immortalized MEPM cells maintain migration ability

**Fig 2:**
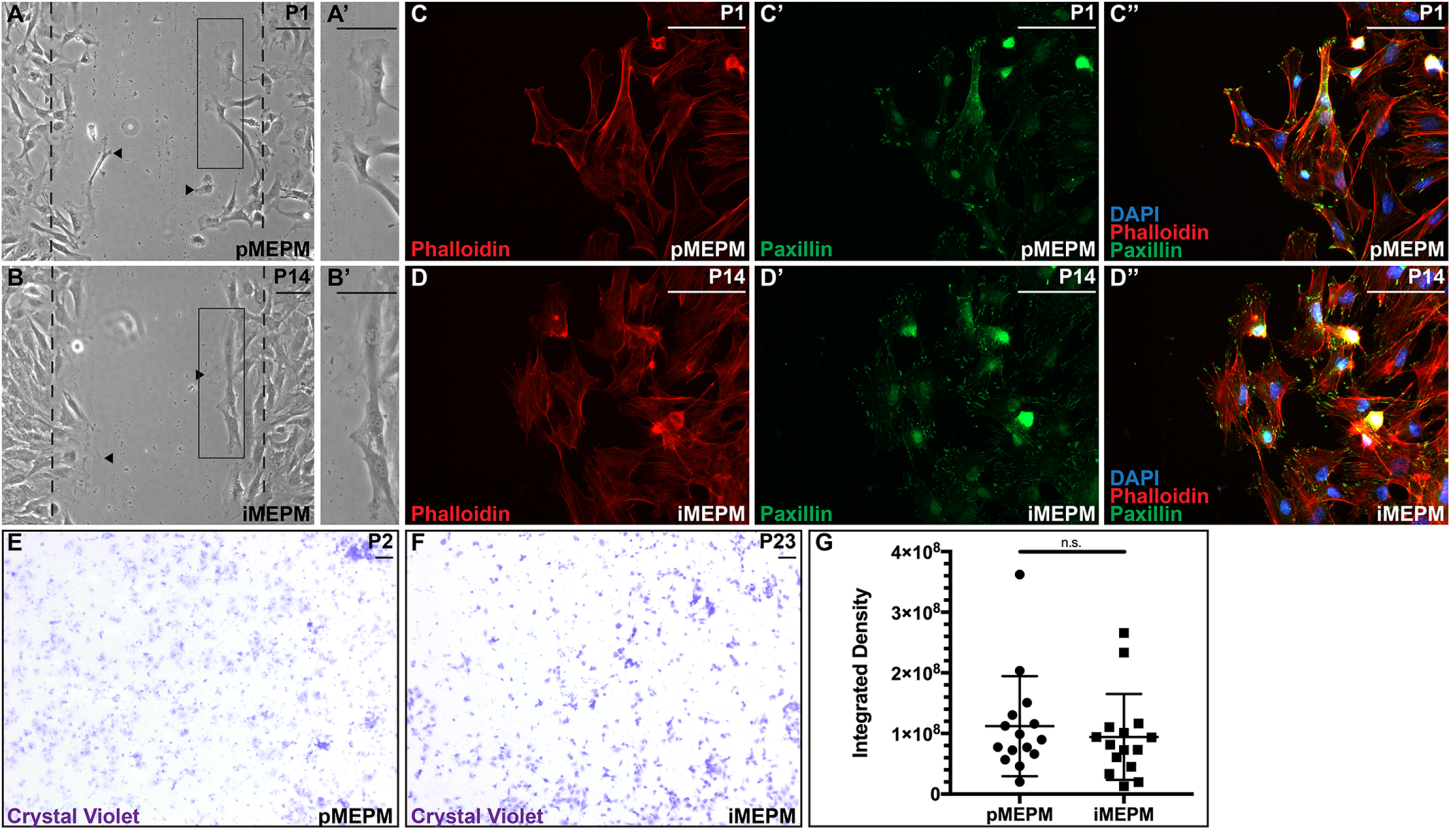
Immortalized MEPM cells maintain migration ability. (A-B’) Primary (A,A’) and immortalized (B,B’) MEPM cells migrate comparable distances following scratching on a fibronectin substrate. Dashed lines indicate the scratch boundary at 0 hours. Black arrowheads denote farthest migration on either side of the scratch. Solid rectangles indicate the frame of the following panel. (C-D”) Expression of phalloidin (red; C,D) and paxillin (green; C’,D’) as assessed by immunofluorescence analyses in passage 1 primary (C-C”) and passage 14 immortalized (D-D”) MEPM cells. Both primary and immortalized MEPM cells at the leading edge display filamentous actin arcs and focal adhesion formation. Nuclei were stained with DAPI (blue; C”,D”). (E-F) Crystal violet staining of primary (E) and immortalized (F) MEPM cells following migration through a porous membrane. Scale bars, 100 μm. (G) Bar graph depicting integrated densities revealing comparable migration of passage 2 primary and passage 23 immortalized MEPM cells in Transwell assays. Data are presented as mean ± SEM. n.s., not significant.

To examine whether immortalized MEPM cells maintain the ability of primary MEPM cells to migrate on an extracellular matrix substrate [10], passage 1 primary MEPM cells and passage 14 immortalized MEPM cells were seeded onto glass coverslips coated with fibronectin. Once cells reached relative confluence, a scratch was introduced with a pipet tip and the cells were allowed to migrate across the wound in medium containing 10% FBS. Photographs taken 6 hours after scratching revealed that the two cell types migrated comparable distances into the wound area (Fig 2A and 2B), with noticeable membrane ruffling and cellular projections at the leading edge (Fig 2A’ and 2B’). Immunofluorescence analyses of these migrating cells demonstrated that both primary and immortalized MEPM cells at the leading edge displayed filamentous actin arcs and focal adhesion formation, highlighted by phalloidin and paxillin staining, respectively (Fig 2C–2D”). Primary MEPM cells have also previously been shown to migrate towards a source of PDGF-AA in Transwell assays [10]. To examine the migration properties of immortalized MEPM cells in this same assay, cells were seeded onto fibronectin-coated polyethylene terephthalate membranes with 8 μm pores in medium containing 0.1% FBS and allowed to migrate into a reservoir with medium containing 10% FBS supplemented with 10 ng/mL PDGF-AA. After 4 hours, migrated cells were stained with crystal violet and quantified, revealing that a comparable number of passage 2 primary and passage 23 immortalized MEPM cells migrated through the pores (112042444 ± 21300234 and 94298626 ± 18275243 integrated densities, respectively; p = 0.5325) (Fig 2E–2G).

### Expression of broad and middle palatal mesenchyme markers is similar between primary and immortalized MEPM cells

**Fig 3:**
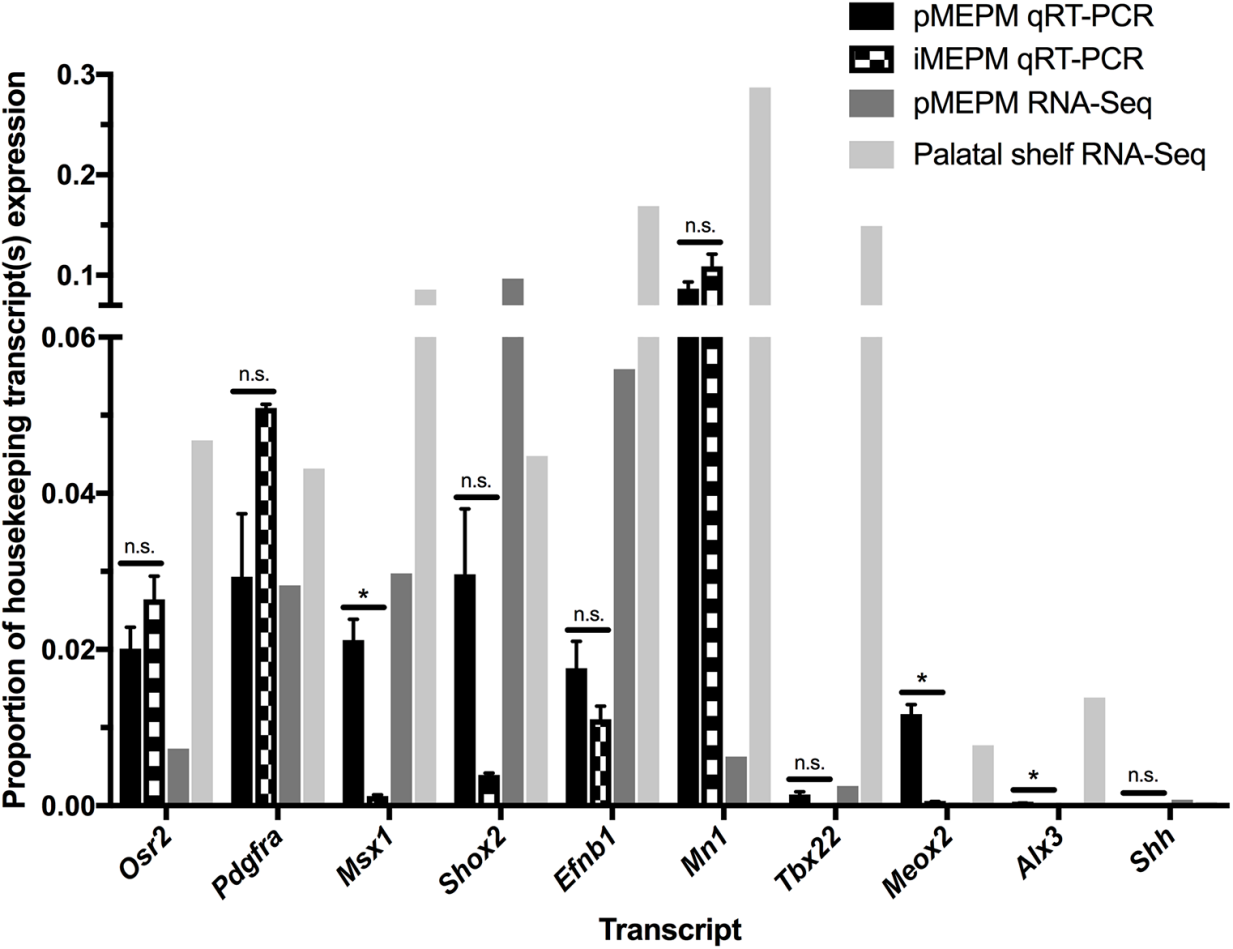
Expression of palatal shelf transcripts is similar between primary and immortalized MEPM cells. Bar graph depicting qRT-PCR values revealing reduced expression of *Msx1*, *Meox2* and *Alx3* in passage 13 immortalized MEPM cells as compared to their passage 1 primary counterparts and similar expression of *Osr2*, *Pdgfra*, *Shox2*, *Efnb1*, *Mn1*, *Tbx22* and *Shh* across the two cell types. qRT-PCR values are normalized to *B2m*. qRT-PCR data are presented as mean ± SEM. n.s., not significant. *, p<0.05. E13.5 primary MEPM RNA-sequencing FPKM (fragments per kilobase of transcript per million mapped reads) values [12] are normalized to *B2m* and E13.5 palatal shelf RNA-sequencing RPKM (reads per kilobase of transcript per million mapped reads) values (www.facebase.org, accession FB00000278) [33] are represented as an average of normalization to *Actb*, *Hsp90ab1* and *Ppia*.

We next performed quantitative real-time PCR (qRT-PCR) to compare the expression of various transcripts expressed during palatal shelf development between primary and immortalized MEPM cells derived from E13.5 embryos (Fig 3). Both primary (passage 1) and immortalized (passage 13) MEPM cells robustly expressed the broad palatal mesenchyme markers *Osr2* [23,24] (0.02011% ± 0.002750% and 0.02640% ± 0.002979% of *B2m*, respectively; p = 0.1960) and *Pdgfra* [25] (0.02930% ± 0.008067% and 0.05093% ± 0.0004891% of *B2m*, respectively; p = 0.1150) at similar levels across the two cell types. While primary and immortalized MEPM cells expressed comparable levels of the anterior palatal mesenchyme markers *Shox2* [26–28] (0.02962% ± 0.008413% and 0.003942% ± 0.0002405% of *B2m*, respectively; p = 0.09260) and *Efnb1* [13] (0.01759% ± 0.003464% and 0.01105% ± 0.001707% of *B2m*, respectively; p = 0.1915), immortalized MEPM cells demonstrated a significant decrease in expression of the extreme anterior palatal mesenchyme marker *Msx1* [28,29] as compared to their primary counterparts (0.02122% ± 0.002663% and 0.001229% ± 0.000146% of *B2m*, respectively; p = 0.01710). Expression of the middle-posterior palatal mesenchyme markers *Mn1* [30] (0.08664% ± 0.006551% and 0.1089% ± 0.01214% of *B2m*, respectively; p = 0.2033) and *Tbx22* [30] (0.001419% ± 0.0003531% and 3.023e^-6^% ± 8.680e^-7^% of *B2m*, respectively; p = 0.0569) were similar across the two cell types. The expression of the posterior palatal mesenchyme marker *Meox2* [28,31] was significantly reduced in immortalized MEPM cells relative to levels in primary MEPM cells (0.01173% ± 0.001221% and 0.0004858% ± 3.992e^-5^% of *B2m*, respectively; p = 0.0115). Finally, expression of the medial nasal process marker *Alx3* [10,32] was measured at very low levels in both cells types (0.0002888% ± 3.855e^-5^% and 6.045e^-6^% ± 1.281e^-6^% of *B2m*, respectively; p = 0.0180), while the anterior palatal epithelium marker *Shh* [29] was virtually undetectable (1.548e^-6^% ± 9.301e^-7^% and 3.131e^-7^% ± 2.122e^-7^% of *B2m*, respectively; p = 0.3143) (Fig 3). These same trends were detected in semi-quantitative PCR analysis of the expression profiles of primary (passage 1) MEPM cells compared to earlier passage (passage 3 and 6) immortalized MEPM cells. Specifically, we detected high and comparable expression of *Osr2* and *Pdgfra*, reduced expression of *Msx1* and *Shox2* in the immortalized MEPM cells and little or no expression of *Tbx22*, *Meox2*, *Alx3* and *Shh* in both cell types (data not shown). Taken together, our expression analyses reveal that immortalized MEPM cells exhibit decreased expression of extreme anterior and posterior palatal mesenchyme markers as compared to their wild-type counterparts, while maintaining comparable expression of broad and middle palatal mesenchyme markers. Similar to primary MEPM cells, immortalized MEPM cells display virtually no expression from contaminating medial nasal process mesenchyme or palatal epithelium.

We subsequently compared the expression profiles of E13.5 immortalized MEPM cells from our qRT-PCR analyses to RNA-sequencing data from E13.5 primary MEPM cells [12] normalized to *B2m* and E13.5 palatal shelves (www.facebase.org, accession FB00000278) [33] normalized to the housekeeping genes *Actb*, *Hsp90ab1* and *Ppia* (Fig 3). With the exception of *Shox2*, the expression of palatal shelf transcripts across the RNA-sequencing datasets tended to be lower in primary MEPM cells than in palatal shelves (Fig 3). Importantly, the expression levels of these same transcripts in E13.5 immortalized MEPM cells were often detected within an order of magnitude of the levels observed in one or both of the RNA-sequencing datasets (in the cases of *Osr2*, *Pdgfra*, *Efnb1*, *Mn1* and *Meox2*) and/or at levels intermediate between those detected in the E13.5 primary MEPM versus palatal shelf samples (in the cases of *Osr2*, *Mn1*, *Meox2* and *Alx3*) (Fig 3).

As highlighted above, the palatal shelves have well-documented molecular heterogeneity along both the medial-lateral and anterior-posterior axes [27,28,34]. Moreover, bromodeoxyuridine (BrdU) labeling experiments have revealed increased mesenchymal proliferation rates in the medial (nasal) versus lateral (oral) palatal shelves at E13.5 [24]. While a similar BrdU incorporation assay indicated equal growth rates of the palatal mesenchyme along the anterior-posterior axis at E13.25 [28], BrdU labeling was not assessed in the extreme anterior and posterior regions of the palatal shelves in this study, nor were the percentages of BrdU-labeled cells quantified. Hence, regional differences in proliferation rates along the anterior-posterior axis of the palatal mesenchyme may exist. As long-term *in vitro* culture would tend to favor cells with increased growth rates, these regional differences in proliferation of the palatal mesenchyme may explain the changes in transcript expression detected between immortalized MEPM cells and either their primary counterparts or intact palatal shelves (Fig 3).

### Immortalized MEPM cells maintain responsiveness to growth factor stimulation

**Fig 4:**
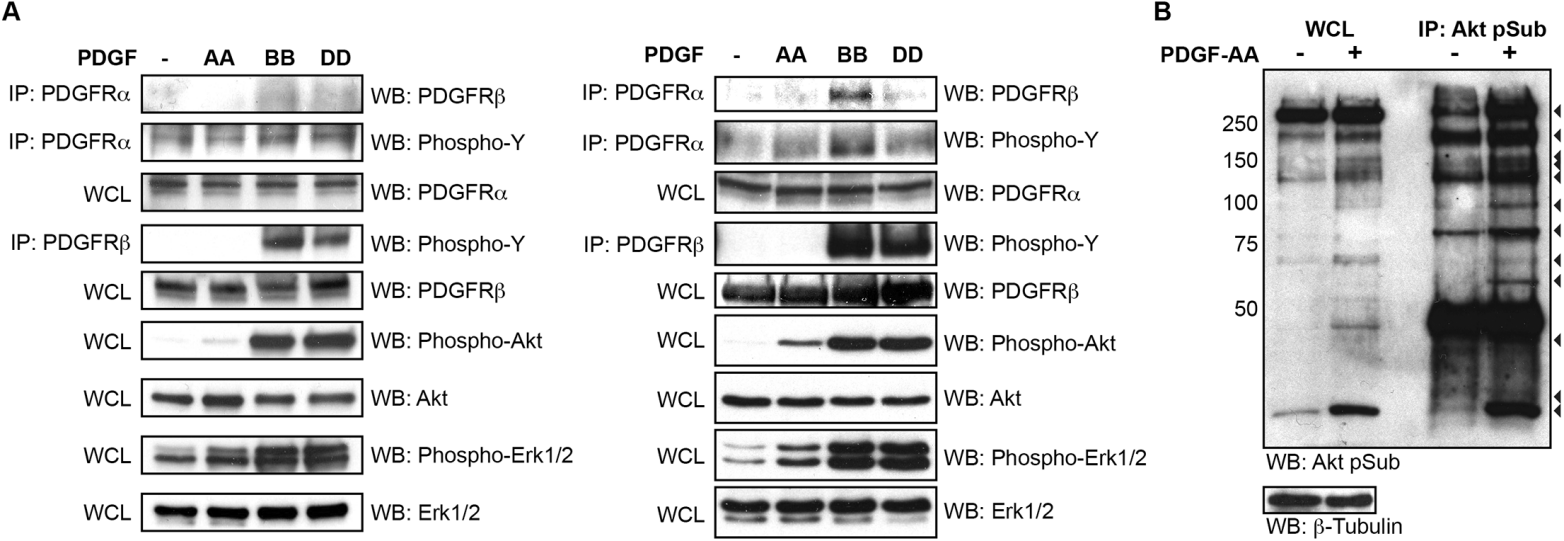
Immortalized MEPM cells maintain responsiveness to growth factor stimulation. (A) Biochemical analysis of PDGFR activation and phosphorylation of downstream signaling molecules in passage 3 primary (left) and passage 12 immortalized (right) MEPM cells following treatment with PDGF-AA, PDGF-BB or PDGF-DD ligand. In both cell types, PDGF-AA treatment led to autophosphorylation of PDGFRα, while stimulation with PDGF-BB and PDGF-DD led to autophosphorylation of PDGFRα and PDGFRβ. PDGF-AA treatment led to modest increases in phospho-Akt and phospho-Erk1/2 levels whereas stimulation with PDGF-BB and PDGF-DD generated robust increases in the levels of each phosphoprotein in both primary and immortalized MEPM cells. (B) Biochemical analysis of PDGF-AA-dependent Akt target phosphorylation in passage 9 immortalized MEPM cells. IP, immunoprecipitation; WCL, whole cell lysate; WB, Western blot.

Primary MEPM cells have previously been shown to be responsive to stimulation with numerous growth factors, such as preclustered ephrin-B1-Fc, PDGF-AA and Fgf1 [10–14]. To examine whether immortalized MEPM cells have similar biochemical profiles to primary MEPM cells, we first examined the ability of various PDGF ligands to induce PDGFR dimerization as well as the phosphorylation of the intracellular signaling proteins Akt and Erk1/2, both of which have demonstrated roles downstream from PDGFR activation during craniofacial development [12,14,35], in each setting. Receptor signaling was stimulated with 10 ng/mL PDGF-AA, PDGF-BB, or PDGF-DD ligand for 15 minutes following overnight starvation in medium containing 0.1% FBS. Immunoprecipitation experiments revealed the presence of PDGFRα/β heterodimers predominantly in response to PDGF-BB treatment in both primary (Fig 4A, left) and immortalized (Fig 4A, right) MEPM cells, consistent with our previous findings [36]. Western blotting with an anti-phosphotyrosine antibody demonstrated that PDGF-AA treatment resulted in autophosphorylation of PDGFRα, while stimulation with PDGF-BB and PDGF-DD led to autophosphorylation of both PDGFRα and PDGFRβ in the two cell types (Fig 4A). Moreover, Western blot analysis of whole cell lysates revealed modest increases in the levels of phospho-Akt and phospho-Erk1/2 over baseline levels upon PDGF-AA treatment and robust increases in the levels of each phosphoprotein in response to stimulation with PDGF-BB and PDGF-DD in both primary (Fig 4A, left) and immortalized (Fig 4A, right) MEPM cells.

We previously demonstrated that PDGF-AA treatment of primary MEPM cells leads to the PI3K-mediated phosphorylation of multiple Akt target proteins [14]. To examine whether this same signaling axis is active in immortalized MEPM cells, we immunoprecipitated Akt targets from untreated and PDGF-AA-treated immortalized MEPM lysates using an anti-Akt phosphosubstrate antibody [37,38]. Subsequent Western blotting with the same antibody revealed at least 12 protein bands with increased intensity upon ligand stimulation (Fig 4B), with a strikingly similar band pattern to that observed in primary MEPM cells [14]. Immortalized MEPM cells thus maintain similar responsiveness to growth factor stimulation as primary MEPM cells, as evidenced by receptor tyrosine kinase dimerization and the phosphorylation of intracellular signaling molecules in response to various PDGF ligands. Indeed, given the increased intensity of bands observed in Western blots of immortalized versus primary MEPM lysates (Fig 4) [14], the former cell type appears to exhibit increased sensitivity to growth factor stimulation over their primary counterparts.

While the method of immortalization utilized here, namely derivation of cells from *Cdkn2a*^*-/-*^ embryos, was chosen based on ease and demonstrated success with a wide range of cell types, alternative techniques exist with unique benefits and drawbacks. One such method is immortalization through transfection or retroviral transduction of an oncogene, which abrogates the time required to generate *Cdkn2a*-deficient mice or embryos. However, disadvantages of this approach have been noted with the commonly used oncogenes SV40 large T antigen and dominant-negative p53, including *in vitro* clonal evolution and impaired differentiation in the former case and a tendency for aneuploidy in the latter [17,39,40]. Improvements of this method have been reported in cases of retroviral transduction of a floxed SV40 large T antigen expression cassette that allows for reversible immortalization [41]. This approach was recently used to generate immortalized MEFs that, upon expression of Cre recombinase, retained the ability to differentiate into multiple lineages [42].

In conclusion, we have generated an immortalized MEPM cell line that has maintained the morphology, migration ability, transcript expression and responsiveness to exogenous growth factors of primary MEPM cells. We anticipate that use of this cell line will facilitate high-throughput, large-scale modeling of palatogenesis *in vitro*. Moreover, introduction of the *Cdkn2a*^*-*^ allele used here onto mutant mouse model backgrounds in which the palatal shelves are reduced in size and/or difficult to culture will allow for expansion of these mutant cells, likely enabling mechanistic studies that have not been possible using primary culture.
